# Bis(4,4′-bipyridinium) dodeca­tungsto­silicate 4,4′-bipyridine hexa­hydrate

**DOI:** 10.1107/S1600536808027566

**Published:** 2008-09-06

**Authors:** Feng-Xia Ma, Quan Zhao

**Affiliations:** a Jilin Agricultural Science and Technology College, Jilin 132101, People’s Republic of China; b Northeast Forestry University, People’s Republic of China

## Abstract

The title compound, (C_10_H_10_N_2_)_2_[SiW_12_O_40_]·C_10_H_8_N_2_·6H_2_O or (4,4′-bipyH_2_)_2_[SiW_12_O_40_].(4,4′-bipy)·6H_2_O (4,4′-bipy is 4,4′-bipyridine), was prepared under hydro­thermal conditions. The asymmetric unit contains a discrete Keggin-type [SiW_12_O_40_]^4−^ anion (located on a twofold axis), one 4,4′-bipy (located on a twofold axis), two (4,4′-bipyH_2_)^2+^ cations and six uncoordinated water mol­ecules. The polyoxoanion is constructed from a central SiO_4_ tetra­hedron which shares its O atoms with four trinuclear W_3_O_13_ groups, each of which is made up of three edge-sharing WO_6_ octa­hedra. The water mol­ecules and [SiW_12_O_40_]^4−^ anions are linked through hydrogen bonds.

## Related literature

For related literature, see: Hill (1998 ▶); Kurth *et al.* (2001 ▶); Misono (1987 ▶); Pope (1983 ▶). H_4_SiW_12_O_40_.*n*H_2_O was prepared according to literature procedures (Rocchiccioli-Deltcheff *et al.*, 1983 ▶).
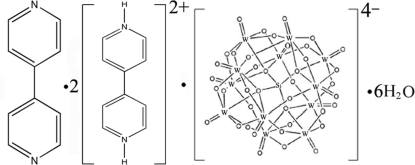


## Experimental

### 

#### Crystal data

(C_10_H_10_N_2_)_2_[SiW_12_O_40_]·C_10_H_8_N_2_·6H_2_O
*M* *_r_* = 3454.85
Monoclinic, 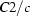

*a* = 15.491 (5) Å
*b* = 18.096 (5) Å
*c* = 20.921 (5) Å
β = 100.834 (5)°
*V* = 5760 (3) Å^3^
*Z* = 4
Mo *K*α radiation
μ = 23.99 mm^−1^
*T* = 293 (2) K
0.15 × 0.12 × 0.10 mm

#### Data collection

Rigaku R-AXIS RAPID diffractometer
Absorption correction: multi-scan (*ABSCOR*; Higashi, 1995 ▶) *T*_min_ = 0.041, *T*_max_ = 0.095
15853 measured reflections
5652 independent reflections
5214 reflections with *I* > 2σ(*I*)
*R*_int_ = 0.042

#### Refinement

*R*[*F* ^2^ > 2σ(*F* ^2^)] = 0.039
*wR*(*F* ^2^) = 0.101
*S* = 1.10
5652 reflections
455 parameters
8 restraints
H atoms treated by a mixture of independent and constrained refinement
Δρ_max_ = 3.60 e Å^−3^
Δρ_min_ = −3.50 e Å^−3^

### 

Data collection: *PROCESS-AUTO* (Rigaku, 1998 ▶); cell refinement: *PROCESS-AUTO*; data reduction: *PROCESS-AUTO*; program(s) used to solve structure: *SHELXS97* (Sheldrick, 2008 ▶); program(s) used to refine structure: *SHELXL97* (Sheldrick, 2008 ▶); molecular graphics: *SHELXTL-Plus* (Sheldrick, 2008 ▶); software used to prepare material for publication: *SHELXL97*.

## Supplementary Material

Crystal structure: contains datablocks global, I. DOI: 10.1107/S1600536808027566/rk2097sup1.cif

Structure factors: contains datablocks I. DOI: 10.1107/S1600536808027566/rk2097Isup2.hkl

Additional supplementary materials:  crystallographic information; 3D view; checkCIF report

## Figures and Tables

**Table 1:** Hydrogen-bond geometry (Å, °)

*D*—H⋯*A*	*D*—H	H⋯*A*	*D*⋯*A*	*D*—H⋯*A*
O1*W*—H1*A*⋯O3*W*	0.89 (10)	2.15 (11)	2.699 (11)	119 (7)
O1*W*—H1*B*⋯O2*W*	0.81 (9)	2.31 (9)	2.749 (12)	115 (10)
O1*W*—H1*B*⋯O20	0.81 (9)	2.54 (11)	2.844 (10)	104 (8)
N1—H1*N*⋯O2*W*	0.78 (9)	1.97 (9)	2.754 (12)	172 (14)
O3*W*—H3*A*⋯O18	0.91 (9)	2.24 (10)	2.878 (10)	127 (9)

**Fig. 1.**
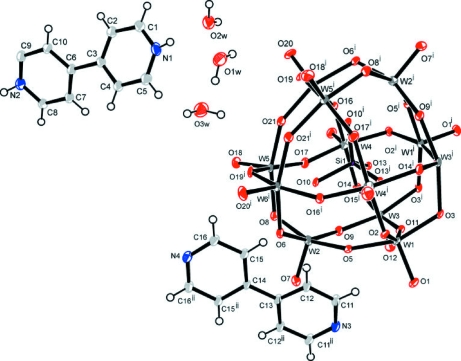
A view of the structure of the title compound. Displacement ellipsoids are drawn at the 50% probability level. H atoms are presented as a small spheres of arbitraryv radius. Symmetry codes: i = -x+2, y, -z+3/2; ii = -x+1, y, -z+3/2.

## supplementary crystallographic information

### Comment

Polyoxometalates (*POM*s) have been known for more than 200 years, but continue to receive attention due to their versatile structures and applications in medicine, materials science, catalysis (Misono, 1987; Pope, 1983; Hill, 1998).

The structure of the title compound is built from one independent 4,4'–bipyridines (4,4'-bipy) (placed on twofold axis by N3/C13/C14/N4) and two protonated 4,4'–bipyridines (4,4'-bipyH_2_)^2+^, and Keggin–type anion [SiW_12_O_40_]^4-^ (placed on twofold axis) and six water molecules (Fig. 1). In the well known Keggin structure, there are 12 WO_6_–octahedra and one SiO_4_–tetrahedron. The 12 WO_6_–octahedra can be categorized into four W_3_O_13_ trinuclear groups, each of which is made of three edge–sharing WO_6_–octahedra and are joined to each other by sharing corners. The SiO_4_–tetrahedron is located in the centre of the polyoxoanion by sharing its O atoms with the four W_3_O_13_–groups. In the Keggin anion, the Si—O and W—O distances as well as the corresponding angles are very similar to those of H_4_SiW_12_O_40_ (Kurth *et al.*, 2001). The water molecules and [SiW_12_O_40_]^4-^ anions are linked through hydrogen bonds. It can be seen that all of the H atoms come from water molecules. The O atoms, which come from the [SiW_12_O_40_]^4-^ anions, are also involved in hydrogen bonds and play the role of acceptors.

### Experimental

The H_4_SiW_12_O_40_^.^nH_2_O was prepared according to the method given by Rocchiccioli-Deltcheff *et al.*, (1983). The starting mixture of H_4_SiW_12_O_40_^.^nH_2_O (0.302 g), 4,4'-bipy (0.026 g), and H_2_O (10 ml) was adjusted to pH = 5.6 by addition of 2 mol.*L*^-1^ NaOH under stirring for 30 min. The final solution was transferred into a 25 ml teflon lined autoclave and was heated at 453 K for 96 h. Then, the autoclave was cooled with a rate of 10 K.h^-1^ to room temperature. Black block–like crystals were filtered off, washed with distilled water and dried at ambient temperature (45% yield on W).

### Refinement

All H atoms on C atoms were poisitioned geometrically and refined as riding atoms, with C—H = 0.93Å and *U*_iso_ = 1.2*U*_eq_(C). The H atoms bonded to N atom and O atoms of water molecules were located in a difference Fourier map and refined isotropically. In the final Fourier map, the highest peak is 0.85Å away from W3 and the deepest hole is 0.55Å from W4.

### Crystal data

**Table:**

(C_10_H_10_N_2_)_2_[SiW_12_O_40_]·C_10_H_8_N_2_·6H_2_O	*F*(000) = 6128
*M**_r_* = 3454.85	*D*_x_ = 3.984 Mg m^−^^3^
Monoclinic, *C*2/*c*	Mo *K*α radiation, λ = 0.71069 Å
Hall symbol: -C 2yc	Cell parameters from 5214 reflections
*a* = 15.491 (5) Å	θ = 1.8–26.0°
*b* = 18.096 (5) Å	µ = 23.99 mm^−^^1^
*c* = 20.921 (5) Å	*T* = 293 K
β = 100.834 (5)°	Block, black
*V* = 5760 (3) Å^3^	0.15 × 0.12 × 0.10 mm
*Z* = 4	

### Data collection

**Table:**

Rigaku R-AXIS RAPID diffractometer	5652 independent reflections
Radiation source: Rotor target	5214 reflections with *I* > 2σ(*I*)
graphite	*R*_int_ = 0.042
Detector resolution: 10.0 pixels mm^-1^	θ_max_ = 26.0°, θ_min_ = 1.8°
ω scans	*h* = −19→14
Absorption correction: multi-scan (ABSCOR; Higashi, 1995)	*k* = −22→22
*T*_min_ = 0.041, *T*_max_ = 0.095	*l* = −23→25
15853 measured reflections	

### Refinement

**Table:**

Refinement on *F*^2^	Primary atom site location: structure-invariant direct methods
Least-squares matrix: full	Secondary atom site location: difference Fourier map
*R*[*F*^2^ > 2σ(*F*^2^)] = 0.039	Hydrogen site location: inferred from neighbouring sites
*wR*(*F*^2^) = 0.101	H atoms treated by a mixture of independent and constrained refinement
*S* = 1.10	*w* = 1/[σ^2^(*F*_o_^2^) + (0.049*P*)^2^ + 129.9363*P*] where *P* = (*F*_o_^2^ + 2*F*_c_^2^)/3
5652 reflections	(Δ/σ)_max_ = 0.002
455 parameters	Δρ_max_ = 3.60 e Å^−^^3^
8 restraints	Δρ_min_ = −3.50 e Å^−^^3^

### Special details

**Table:**

Geometry. All s.u.'s (except the s.u. in the dihedral angle between two l.s. planes) are estimated using the full covariance matrix. The cell s.u.'s are taken into account individually in the estimation of s.u.'s in distances, angles and torsion angles; correlations between s.u.'s in cell parameters are only used when they are defined by crystal symmetry. An approximate (isotropic) treatment of cell s.u.'s is used for estimating s.u.'s involving l.s. planes.
Refinement. Refinement of *F*^2^ against ALL reflections. The weighted *R*–factor *wR* and goodness of fit *S* are based on *F*^2^, conventional *R*–factors *R* are based on *F*, with *F* set to zero for negative *F*^2^. The threshold expression of *F*^2^ > σ(*F*^2^) is used only for calculating *R*–factors(gt) *etc*. and is not relevant to the choice of reflections for refinement. *R*–factors based on *F*^2^ are statistically about twice as largeas those based on *F*, and *R*–factors based on ALL data will be even larger.

### Fractional atomic coordinates and isotropic or equivalent isotropic displacement parameters (Å^2^)

**Table:**

	*x*	*y*	*z*	*U*_iso_*/*U*_eq_	
Si1	1.0000	0.14417 (15)	0.7500	0.0055 (5)	
W1	0.98109 (2)	0.004870 (18)	0.865205 (16)	0.01358 (10)	
W2	0.81326 (2)	0.150489 (19)	0.823667 (17)	0.01562 (10)	
W3	0.83728 (2)	0.008359 (18)	0.701365 (16)	0.01280 (10)	
W4	0.86857 (2)	0.136541 (19)	0.593364 (17)	0.01711 (10)	
W5	0.83464 (2)	0.280029 (18)	0.712749 (16)	0.01327 (10)	
W6	1.03445 (2)	0.282564 (18)	0.639007 (16)	0.01417 (10)	
C1	0.7254 (7)	0.6611 (5)	0.4178 (5)	0.0264 (14)	
H1	0.7714	0.6664	0.3953	0.032*	
C2	0.6599 (7)	0.7125 (5)	0.4101 (5)	0.0254 (13)	
H2	0.6598	0.7520	0.3816	0.031*	
C3	0.5934 (6)	0.7045 (5)	0.4454 (5)	0.0210 (13)	
C4	0.5952 (7)	0.6445 (5)	0.4877 (5)	0.0223 (13)	
H4	0.5515	0.6386	0.5123	0.027*	
C5	0.6624 (7)	0.5948 (5)	0.4922 (5)	0.0266 (13)	
H5	0.6649	0.5544	0.5200	0.032*	
C6	0.5183 (6)	0.7587 (5)	0.4376 (5)	0.0210 (13)	
C7	0.4350 (6)	0.7337 (5)	0.4437 (5)	0.0219 (13)	
H7	0.4262	0.6843	0.4530	0.026*	
C8	0.3650 (7)	0.7838 (5)	0.4357 (5)	0.0228 (14)	
H8	0.3090	0.7681	0.4396	0.027*	
C9	0.4582 (6)	0.8799 (5)	0.4160 (5)	0.0240 (14)	
H9	0.4648	0.9297	0.4069	0.029*	
C10	0.5303 (7)	0.8325 (5)	0.4228 (5)	0.0227 (13)	
H10	0.5850	0.8498	0.4175	0.027*	
C11	0.5736 (6)	−0.0110 (5)	0.7442 (4)	0.0195 (13)	
H11	0.6235	−0.0379	0.7401	0.023*	
C12	0.5755 (6)	0.0660 (5)	0.7443 (4)	0.0174 (12)	
H12	0.6269	0.0908	0.7406	0.021*	
C13	0.5000	0.1061 (7)	0.7500	0.0159 (14)	
C14	0.5000	0.1881 (6)	0.7500	0.0146 (14)	
C15	0.5682 (6)	0.2278 (5)	0.7320 (4)	0.0174 (12)	
H15	0.6147	0.2029	0.7193	0.021*	
C16	0.5676 (6)	0.3045 (5)	0.7328 (4)	0.0206 (13)	
H16	0.6141	0.3307	0.7214	0.025*	
N1	0.7246 (6)	0.6045 (5)	0.4565 (4)	0.0265 (13)	
H1N	0.770 (5)	0.584 (6)	0.461 (6)	0.040*	
N2	0.3794 (6)	0.8539 (4)	0.4226 (4)	0.0252 (14)	
H2N	0.334 (5)	0.875 (6)	0.419 (6)	0.038*	
N3	0.5000	−0.0457 (6)	0.7500	0.0192 (16)	
N4	0.5000	0.3407 (6)	0.7500	0.0192 (16)	
O1	0.9447 (4)	−0.0617 (3)	0.9112 (3)	0.0163 (12)	
O2	1.0599 (4)	0.0597 (3)	0.9308 (3)	0.0120 (10)	
O3	1.0822 (4)	−0.0439 (3)	0.8440 (3)	0.0122 (10)	
O5	0.8959 (4)	0.0815 (3)	0.8665 (3)	0.0116 (9)	
O6	0.8667 (4)	0.2281 (3)	0.8797 (3)	0.0134 (9)	
O7	0.7236 (4)	0.1323 (3)	0.8568 (3)	0.0165 (11)	
O8	0.7635 (4)	0.2270 (3)	0.7623 (3)	0.0146 (8)	
O9	0.7957 (4)	0.0824 (3)	0.7520 (3)	0.0141 (8)	
O10	0.9299 (4)	0.1957 (3)	0.7773 (3)	0.0104 (8)	
O11	0.9167 (4)	−0.0197 (3)	0.7808 (3)	0.0135 (9)	
O12	0.7571 (4)	−0.0570 (3)	0.6969 (3)	0.0198 (12)	
O13	0.9484 (4)	0.0926 (3)	0.6913 (3)	0.0096 (8)	
O14	0.7932 (4)	0.0625 (3)	0.6220 (3)	0.0135 (9)	
O15	0.8026 (4)	0.1562 (3)	0.5197 (3)	0.0184 (12)	
O16	0.9609 (4)	0.2067 (3)	0.5959 (3)	0.0136 (9)	
O17	0.8220 (4)	0.2048 (3)	0.6488 (3)	0.0146 (9)	
O18	0.7599 (4)	0.3442 (3)	0.6770 (3)	0.0198 (12)	
O19	1.1124 (4)	0.3313 (3)	0.7084 (3)	0.0136 (10)	
O20	1.0266 (5)	0.3501 (3)	0.5807 (3)	0.0214 (13)	
O21	0.9400 (4)	0.3087 (3)	0.6807 (3)	0.0159 (9)	
O1W	0.9247 (5)	0.4797 (4)	0.5870 (4)	0.0332 (12)	
H1A	0.890 (7)	0.469 (7)	0.615 (4)	0.050*	
H1B	0.920 (8)	0.457 (6)	0.553 (4)	0.050*	
O2W	0.8790 (5)	0.5277 (4)	0.4605 (4)	0.0319 (13)	
H2A	0.932 (7)	0.547 (6)	0.463 (5)	0.048*	
H2B	0.859 (7)	0.506 (6)	0.427 (4)	0.048*	
O3W	0.7508 (5)	0.4587 (4)	0.5805 (4)	0.0380 (13)	
H3A	0.780 (8)	0.416 (4)	0.594 (5)	0.057*	
H3B	0.763 (9)	0.487 (5)	0.618 (4)	0.057*	

### Atomic displacement parameters (Å^2^)

**Table:**

	*U*^11^	*U*^22^	*U*^33^	*U*^12^	*U*^13^	*U*^23^
Si1	0.0050 (12)	0.0012 (11)	0.0111 (12)	0.000	0.0033 (10)	0.000
W1	0.0161 (2)	0.00723 (17)	0.01860 (19)	0.00094 (13)	0.00648 (14)	0.00361 (12)
W2	0.0143 (2)	0.01192 (18)	0.02205 (19)	0.00164 (13)	0.00714 (14)	−0.00002 (13)
W3	0.0121 (2)	0.00709 (17)	0.01982 (18)	−0.00403 (12)	0.00457 (13)	−0.00200 (12)
W4	0.0176 (2)	0.01453 (19)	0.01848 (19)	0.00010 (14)	0.00146 (14)	0.00044 (13)
W5	0.0137 (2)	0.00674 (17)	0.01943 (18)	0.00445 (12)	0.00332 (13)	0.00175 (12)
W6	0.0179 (2)	0.00714 (17)	0.01819 (18)	−0.00104 (13)	0.00512 (14)	0.00300 (12)
C1	0.022 (3)	0.022 (3)	0.037 (3)	0.000 (2)	0.010 (2)	−0.002 (2)
C2	0.023 (3)	0.019 (2)	0.035 (3)	0.001 (2)	0.008 (2)	0.000 (2)
C3	0.018 (3)	0.017 (2)	0.029 (3)	0.000 (2)	0.007 (2)	−0.002 (2)
C4	0.019 (2)	0.018 (2)	0.031 (2)	0.001 (2)	0.007 (2)	−0.001 (2)
C5	0.023 (3)	0.022 (3)	0.034 (3)	0.001 (2)	0.005 (2)	0.001 (2)
C6	0.020 (3)	0.015 (2)	0.028 (3)	0.002 (2)	0.005 (2)	0.000 (2)
C7	0.020 (3)	0.017 (2)	0.030 (3)	0.003 (2)	0.006 (2)	0.000 (2)
C8	0.019 (3)	0.019 (3)	0.032 (3)	0.003 (3)	0.009 (3)	0.002 (3)
C9	0.023 (3)	0.016 (3)	0.033 (3)	0.002 (3)	0.006 (3)	−0.002 (3)
C10	0.021 (3)	0.016 (2)	0.031 (3)	0.000 (2)	0.007 (2)	0.000 (2)
C11	0.018 (2)	0.012 (2)	0.029 (2)	0.001 (2)	0.003 (2)	−0.002 (2)
C12	0.016 (2)	0.011 (2)	0.025 (2)	0.000 (2)	0.003 (2)	0.000 (2)
C13	0.015 (3)	0.010 (3)	0.022 (3)	0.000	0.002 (2)	0.000
C14	0.014 (3)	0.009 (3)	0.021 (3)	0.000	0.004 (2)	0.000
C15	0.016 (2)	0.011 (2)	0.025 (2)	0.000 (2)	0.004 (2)	0.000 (2)
C16	0.020 (3)	0.014 (2)	0.028 (3)	−0.002 (2)	0.004 (2)	0.002 (2)
N1	0.020 (3)	0.021 (2)	0.038 (3)	0.004 (2)	0.005 (2)	−0.004 (2)
N2	0.023 (3)	0.019 (3)	0.034 (3)	0.006 (2)	0.008 (2)	0.001 (2)
N3	0.018 (3)	0.011 (3)	0.029 (3)	0.000	0.004 (3)	0.000
N4	0.019 (3)	0.013 (3)	0.025 (3)	0.000	0.002 (3)	0.000
O1	0.018 (3)	0.010 (3)	0.023 (3)	0.001 (2)	0.007 (2)	0.007 (2)
O2	0.011 (2)	0.0089 (19)	0.016 (2)	0.0007 (18)	0.0036 (17)	0.0043 (17)
O3	0.013 (2)	0.005 (2)	0.019 (2)	0.0014 (18)	0.0047 (18)	0.0035 (18)
O5	0.0132 (18)	0.0082 (17)	0.0149 (17)	−0.0005 (16)	0.0060 (15)	0.0017 (15)
O6	0.0144 (19)	0.0075 (18)	0.0190 (18)	0.0021 (16)	0.0049 (17)	−0.0011 (16)
O7	0.015 (3)	0.014 (2)	0.021 (2)	0.003 (2)	0.006 (2)	0.000 (2)
O8	0.0131 (17)	0.0107 (16)	0.0201 (16)	0.0028 (15)	0.0037 (15)	0.0016 (15)
O9	0.0138 (16)	0.0097 (15)	0.0194 (15)	0.0000 (14)	0.0050 (14)	−0.0002 (14)
O10	0.0116 (17)	0.0047 (16)	0.0155 (16)	0.0012 (15)	0.0039 (15)	0.0004 (14)
O11	0.0158 (18)	0.0084 (17)	0.0171 (18)	−0.0005 (16)	0.0049 (16)	0.0026 (16)
O12	0.016 (3)	0.014 (3)	0.030 (3)	−0.004 (2)	0.007 (2)	−0.002 (2)
O13	0.0106 (16)	0.0052 (16)	0.0142 (16)	0.0010 (15)	0.0054 (14)	−0.0004 (14)
O14	0.0119 (18)	0.0090 (17)	0.0194 (17)	−0.0019 (16)	0.0025 (16)	−0.0018 (16)
O15	0.019 (3)	0.016 (3)	0.019 (3)	0.002 (2)	0.002 (2)	0.004 (2)
O16	0.0141 (19)	0.0090 (18)	0.0189 (18)	0.0006 (17)	0.0061 (16)	0.0016 (16)
O17	0.0140 (17)	0.0103 (16)	0.0193 (16)	0.0017 (15)	0.0023 (15)	0.0004 (15)
O18	0.022 (3)	0.016 (2)	0.023 (3)	0.009 (2)	0.007 (2)	0.007 (2)
O19	0.015 (2)	0.005 (2)	0.021 (2)	−0.0026 (18)	0.0053 (18)	0.0020 (18)
O20	0.025 (3)	0.015 (3)	0.026 (3)	0.000 (2)	0.008 (2)	0.012 (2)
O21	0.0165 (18)	0.0096 (17)	0.0224 (18)	0.0011 (16)	0.0053 (16)	0.0014 (16)
O1W	0.029 (2)	0.026 (2)	0.044 (2)	−0.001 (2)	0.006 (2)	0.005 (2)
O2W	0.025 (3)	0.026 (3)	0.045 (3)	0.001 (2)	0.008 (2)	−0.003 (2)
O3W	0.032 (3)	0.033 (2)	0.048 (3)	0.001 (2)	0.005 (2)	0.007 (2)

### Geometric parameters (Å, °)

**Table:**

Si1—O10	1.614 (6)	C3—C4	1.397 (13)
Si1—O10^i^	1.614 (6)	C3—C6	1.507 (13)
Si1—O13	1.627 (5)	C4—C5	1.366 (13)
Si1—O13^i^	1.627 (5)	C4—H4	0.9300
W1—O1	1.703 (6)	C5—N1	1.337 (14)
W1—O11	1.911 (5)	C5—H5	0.9300
W1—O5	1.918 (6)	C6—C10	1.391 (13)
W1—O3	1.921 (6)	C6—C7	1.395 (14)
W1—O2	1.931 (6)	C7—C8	1.400 (13)
W1—O13^i^	2.366 (5)	C7—H7	0.9300
W2—O7	1.697 (6)	C8—N2	1.325 (12)
W2—O5	1.889 (5)	C8—H8	0.9300
W2—O6	1.915 (6)	C9—N2	1.340 (13)
W2—O9	1.921 (6)	C9—C10	1.394 (14)
W2—O8	1.945 (6)	C9—H9	0.9300
W2—O10	2.353 (6)	C10—H10	0.9300
W3—O12	1.706 (6)	C11—N3	1.327 (11)
W3—O9	1.894 (6)	C11—C12	1.395 (12)
W3—O14	1.939 (6)	C11—H11	0.9300
W3—O11	1.940 (5)	C12—C13	1.400 (11)
W3—O3^i^	1.948 (6)	C12—H12	0.9300
W3—O13	2.338 (5)	C13—C12^ii^	1.400 (11)
W4—O15	1.719 (6)	C13—C14	1.483 (17)
W4—O2^i^	1.904 (6)	C14—C15^ii^	1.388 (11)
W4—O16	1.907 (6)	C14—C15	1.388 (11)
W4—O17	1.924 (6)	C15—C16	1.387 (12)
W4—O14	1.943 (6)	C15—H15	0.9300
W4—O13	2.325 (5)	C16—N4	1.341 (11)
W5—O18	1.709 (6)	C16—H16	0.9300
W5—O17	1.894 (6)	N1—H1N	0.79 (6)
W5—O8	1.909 (6)	N2—H2N	0.79 (6)
W5—O19^i^	1.935 (6)	N3—C11^ii^	1.327 (11)
W5—O21	1.948 (6)	N4—C16^ii^	1.341 (11)
W5—O10	2.363 (5)	O2—W4^i^	1.904 (6)
W6—O20	1.715 (6)	O3—W3^i^	1.948 (6)
W6—O21	1.898 (6)	O6—W6^i^	1.923 (6)
W6—O16	1.900 (6)	O10—W6^i^	2.341 (5)
W6—O19	1.919 (6)	O13—W1^i^	2.366 (5)
W6—O6^i^	1.923 (6)	O19—W5^i^	1.935 (6)
W6—O10^i^	2.341 (5)	O1W—H1A	0.89 (6)
C1—N1	1.306 (13)	O1W—H1B	0.81 (6)
C1—C2	1.364 (14)	O2W—H2A	0.88 (9)
C1—H1	0.9300	O2W—H2B	0.80 (6)
C2—C3	1.383 (14)	O3W—H3A	0.92 (6)
C2—H2	0.9300	O3W—H3B	0.93 (6)
			
O10—Si1—O10^i^	109.4 (4)	O19—W6—O6^i^	88.7 (2)
O10—Si1—O13	109.3 (3)	O20—W6—O10^i^	170.4 (3)
O10^i^—Si1—O13	109.4 (3)	O21—W6—O10^i^	85.3 (2)
O10—Si1—O13^i^	109.4 (3)	O16—W6—O10^i^	84.6 (2)
O10^i^—Si1—O13^i^	109.3 (3)	O19—W6—O10^i^	73.5 (2)
O13—Si1—O13^i^	110.0 (4)	O6^i^—W6—O10^i^	74.0 (2)
O1—W1—O11	100.7 (3)	N1—C1—C2	120.9 (10)
O1—W1—O5	101.7 (3)	N1—C1—H1	119.5
O11—W1—O5	86.6 (2)	C2—C1—H1	119.5
O1—W1—O3	100.2 (3)	C1—C2—C3	118.5 (10)
O11—W1—O3	89.2 (2)	C1—C2—H2	120.8
O5—W1—O3	158.2 (2)	C3—C2—H2	120.8
O1—W1—O2	101.2 (3)	C2—C3—C4	119.5 (9)
O11—W1—O2	158.0 (2)	C2—C3—C6	120.7 (9)
O5—W1—O2	87.9 (2)	C4—C3—C6	119.8 (9)
O3—W1—O2	88.0 (2)	C5—C4—C3	118.6 (10)
O1—W1—O13^i^	172.0 (2)	C5—C4—H4	120.7
O11—W1—O13^i^	84.5 (2)	C3—C4—H4	120.7
O5—W1—O13^i^	84.6 (2)	N1—C5—C4	119.7 (9)
O3—W1—O13^i^	73.7 (2)	N1—C5—H5	120.2
O2—W1—O13^i^	73.8 (2)	C4—C5—H5	120.2
O7—W2—O5	102.2 (3)	C10—C6—C7	119.7 (9)
O7—W2—O6	100.6 (3)	C10—C6—C3	121.1 (9)
O5—W2—O6	91.0 (2)	C7—C6—C3	119.2 (8)
O7—W2—O9	100.8 (3)	C6—C7—C8	119.3 (9)
O5—W2—O9	86.0 (2)	C6—C7—H7	120.4
O6—W2—O9	158.5 (3)	C8—C7—H7	120.4
O7—W2—O8	98.9 (3)	N2—C8—C7	119.2 (9)
O5—W2—O8	158.8 (3)	N2—C8—H8	120.4
O6—W2—O8	87.4 (2)	C7—C8—H8	120.4
O9—W2—O8	87.8 (2)	N2—C9—C10	120.0 (9)
O7—W2—O10	170.8 (2)	N2—C9—H9	120.0
O5—W2—O10	85.5 (2)	C10—C9—H9	120.0
O6—W2—O10	73.8 (2)	C6—C10—C9	118.4 (9)
O9—W2—O10	84.7 (2)	C6—C10—H10	120.8
O8—W2—O10	73.8 (2)	C9—C10—H10	120.8
O12—W3—O9	101.6 (3)	N3—C11—C12	119.3 (9)
O12—W3—O14	99.7 (3)	N3—C11—H11	120.3
O9—W3—O14	91.1 (2)	C12—C11—H11	120.3
O12—W3—O11	101.7 (3)	C11—C12—C13	120.1 (9)
O9—W3—O11	86.2 (2)	C11—C12—H12	119.9
O14—W3—O11	158.5 (2)	C13—C12—H12	119.9
O12—W3—O3^i^	99.4 (3)	C12^ii^—C13—C12	117.5 (11)
O9—W3—O3^i^	158.7 (2)	C12^ii^—C13—C14	121.2 (6)
O14—W3—O3^i^	89.0 (2)	C12—C13—C14	121.2 (6)
O11—W3—O3^i^	85.9 (2)	C15^ii^—C14—C15	117.5 (11)
O12—W3—O13	171.3 (2)	C15^ii^—C14—C13	121.2 (6)
O9—W3—O13	85.6 (2)	C15—C14—C13	121.2 (5)
O14—W3—O13	74.9 (2)	C16—C15—C14	120.5 (9)
O11—W3—O13	83.6 (2)	C16—C15—H15	119.7
O3^i^—W3—O13	73.9 (2)	C14—C15—H15	119.7
O15—W4—O2^i^	101.0 (3)	N4—C16—C15	120.0 (9)
O15—W4—O16	101.7 (3)	N4—C16—H16	120.0
O2^i^—W4—O16	91.3 (2)	C15—C16—H16	120.0
O15—W4—O17	100.4 (3)	C1—N1—C5	122.8 (9)
O2^i^—W4—O17	158.6 (2)	C1—N1—H1N	109 (9)
O16—W4—O17	85.4 (3)	C5—N1—H1N	127 (9)
O15—W4—O14	97.4 (3)	C8—N2—C9	123.4 (9)
O2^i^—W4—O14	89.5 (2)	C8—N2—H2N	108 (9)
O16—W4—O14	160.3 (2)	C9—N2—H2N	129 (9)
O17—W4—O14	86.7 (2)	C11^ii^—N3—C11	123.6 (11)
O15—W4—O13	171.5 (2)	C16^ii^—N4—C16	121.4 (11)
O2^i^—W4—O13	75.2 (2)	W4^i^—O2—W1	120.5 (3)
O16—W4—O13	86.1 (2)	W1—O3—W3^i^	121.0 (3)
O17—W4—O13	83.5 (2)	W2—O5—W1	151.4 (3)
O14—W4—O13	75.1 (2)	W2—O6—W6^i^	121.3 (3)
O18—W5—O17	102.0 (3)	W5—O8—W2	121.2 (3)
O18—W5—O8	99.6 (3)	W3—O9—W2	151.4 (3)
O17—W5—O8	91.6 (3)	Si1—O10—W6^i^	125.0 (3)
O18—W5—O19^i^	101.0 (3)	Si1—O10—W2	124.4 (3)
O17—W5—O19^i^	156.7 (2)	W6^i^—O10—W2	90.93 (19)
O8—W5—O19^i^	88.5 (3)	Si1—O10—W5	124.1 (3)
O18—W5—O21	102.2 (3)	W6^i^—O10—W5	91.59 (19)
O17—W5—O21	86.0 (3)	W2—O10—W5	90.8 (2)
O8—W5—O21	158.1 (2)	W1—O11—W3	150.2 (3)
O19^i^—W5—O21	85.3 (2)	Si1—O13—W4	125.0 (3)
O18—W5—O10	171.0 (3)	Si1—O13—W3	124.9 (3)
O17—W5—O10	84.8 (2)	W4—O13—W3	91.37 (19)
O8—W5—O10	74.2 (2)	Si1—O13—W1^i^	123.6 (3)
O19^i^—W5—O10	72.7 (2)	W4—O13—W1^i^	90.46 (18)
O21—W5—O10	84.0 (2)	W3—O13—W1^i^	91.39 (18)
O20—W6—O21	101.0 (3)	W3—O14—W4	118.5 (3)
O20—W6—O16	102.7 (3)	W6—O16—W4	150.9 (3)
O21—W6—O16	87.3 (3)	W5—O17—W4	152.4 (3)
O20—W6—O19	99.3 (3)	W6—O19—W5^i^	122.0 (3)
O21—W6—O19	88.2 (3)	W6—O21—W5	149.3 (3)
O16—W6—O19	158.0 (2)	H1A—O1W—H1B	119 (9)
O20—W6—O6^i^	99.9 (3)	H2A—O2W—H2B	117 (10)
O21—W6—O6^i^	159.0 (2)	H3A—O3W—H3B	102 (6)
O16—W6—O6^i^	87.8 (2)		

Symmetry codes: (i) −*x*+2, *y*, −*z*+3/2; (ii) −*x*+1, *y*, −*z*+3/2.

### Hydrogen-bond geometry (Å, °)

**Table:**

*D*—H···*A*	*D*—H	H···*A*	*D*···*A*	*D*—H···*A*
O1W—H1A···O3W	0.89 (10)	2.15 (11)	2.699 (11)	119 (7)
O1W—H1B···O2W	0.81 (9)	2.31 (9)	2.749 (12)	115 (10)
O1W—H1B···O20	0.81 (9)	2.54 (11)	2.844 (10)	104 (8)
N1—H1N···O2W	0.78 (9)	1.97 (9)	2.754 (12)	172 (14)
O3W—H3A···O18	0.91 (9)	2.24 (10)	2.878 (10)	127 (9)
